# Correlation of cell membrane dynamics and cell motility

**DOI:** 10.1186/1471-2105-12-S13-S19

**Published:** 2011-11-30

**Authors:** Merlin Veronika, Roy Welsch, Alvin Ng, Paul Matsudaira, Jagath C Rajapakse

**Affiliations:** 1 Computation and Systems Biology, Singapore-MIT Alliance, Nanyang Technological University, Singapore 637460; 2 BioInformatics Research Centre, Nanyang Technological University, Singapore 637553; 3 Sloan School of Management, Massachusetts Institute of Technology, Cambridge, MA 02142, USA; 4 Department of Biological Sciences, National University of Singapore, Singapore 117543; 5 Centre for BioImaging Sciences, National University of Singapore, Singapore 117543; 6 Mechanobiology Institute, National University of Singapore, Singapore 117411; 7 Department of Biological Engineering, Massachusetts Institute of Technology, Cambridge, MA 02142, USA

## Abstract

**Background:**

Essential events of cell development and homeostasis are revealed by the associated changes of cell morphology and therefore have been widely used as a key indicator of physiological states and molecular pathways affecting various cellular functions via cytoskeleton. Cell motility is a complex phenomenon primarily driven by the actin network, which plays an important role in shaping the morphology of the cells. Most of the morphology based features are approximated from cell periphery but its dynamics have received none to scant attention. We aim to bridge the gap between membrane dynamics and cell states from the perspective of whole cell movement by identifying cell edge patterns and its correlation with cell dynamics.

**Results:**

We present a systematic study to extract, classify, and compare cell dynamics in terms of cell motility and edge activity. Cell motility features extracted by fitting a persistent random walk were used to identify the initial set of cell subpopulations. We propose algorithms to extract edge features along the entire cell periphery such as protrusion and retraction velocity. These constitute a unique set of multivariate time-lapse edge features that are then used to profile subclasses of cell dynamics by unsupervised clustering.

**Conclusions:**

By comparing membrane dynamic patterns exhibited by each subclass of cells, correlated trends of edge and cell movements were identified. Our findings are consistent with published literature and we also identified that motility patterns are influenced by edge features from initial time points compared to later sampling intervals.

## Background

Cellular populations exhibit phenotypic heterogeneity across various physiological and pathological processes. The causative factors range from biological noise to complex distinct states of cell functions. Different approaches have been reported to study cellular heterogeneity from different fronts. Morphological responses to perturbations in cellular environments have been characterized by patterns of signaling marker colocalization from high content images [1]. Cellular heterogeneity through FACS (fluorescence activated cell sorting) has been captured to provide a large number of cell read outs, but without any spatial information [2]. Earlier studies have profiled cell subpopulations from fluorescent images by computing dynamic features of the cells along with static features by using unsupervised clustering [3]. Cellular morphology is a highly dynamic entity and time-lapse high-content imaging of cells provides an unprecedented opportunity to understand the mechanisms of morphodynamics. Morphodynamics is defined as a correlation of cell morphology and the underlying functional activity with respect to time [4]. This concept has enabled the discovery of functionality of specific biomolecules and demanded new techniques for interpretability, accuracy, and speed. Extensive research has been performed in understanding and application of morphodynamics of cell edges. High throughput analysis of cell morphodynamics has been used to discover functions of specific proteins [5]. A series of studies using quantitative fluorescent speckle microscopy have revealed the power of computer assisted high throughput analysis of time-lapse microscopy images: an analysis of the number of speckles suggested distinct regulation of actin polymerization-depolymerization dynamics in different intracellular regions [6,7]. The ratio of protrusive to inactive cell perimeter has been used as the measure of cell edge activity [8]. Difference of the cell membrane boundary was reported in the study of cell spread dynamics [9] and its role in actin transport for protruding lamellipodia [10], formation of filopodia downstream of SCAR (Suppressor of cAMP receptor) [11], and the role of cofilin as a promoter of actin polymerization leading to protrusion [12]. Alternatively protrusion rates are measured at multiple locations of the cell boundary. The morphological changes have been studied by placing markers in the cell boundary at regular intervals and tracking their displacement in orthogonal directions to the cell boundary [13]. Instead of direct displacement of tracking, cell boundaries can be analyzed with kymographs [14]. This technique involves high resolution time-lapse microscopy to capture subcellular motion which is widely used for relatively small sample sizes due to highly magnified imaging and for relatively short periods of time. However, these approaches are not suitable for high throughput applications due to computational complexity compounded by elaborate cell shapes and its ever changing dynamics.

In this work, we propose novel morphodynamics concepts to quantify the relationship between whole cell movement and edge dynamics. Whole cell movement as a function of space and time and its possible influence on protrusion retraction dynamics have not been studied in detail. Heterogeneous populations exhibiting characteristic protrusion and retraction patterns have been completely exploited by us in order to identify possible correlations with motility features. Such information is helpful in determining overall motility functions of cells in collective migration. Cell membrane movements are extracted and protrusion/retraction dynamics along the cell edges at different time points were obtained to correlate with whole cell motility features. An approach to extract such patterns from heterogeneous cell populations is presented. Our experiments show that the cells with similar kinetic profiles display different edge movements and that features observed in initial time points have profound influence in determining the type of motility patterns as the cell adapts to its motion.

## Results and discussion

### Dataset

Cells used in this experiment were mouse macrophage cell lines IC-21 (American Type Culture Collection (ATCC) TIB-186) treated with solvent DMSO (Dimethyl sulphoxide). Cells were observed over a period of 120 minutes and 12-bit images with 0.5 *µ*m^2^ pixels were collected using Cellomics KineticScan at every 10 minutes giving a total of 12 snapshots. Data and statistical analysis were implemented in MATLAB R2008a (The Mathworks, Inc., USA) and R project [15].

### Cell identification and tracking

Cells are bright objects protruding from a relatively uniform dark background in microscopic images. The purpose of segmentation is to identify cells accurately in an automated manner. Segmentation algorithms cluster image pixels based on their features into two groups representing objects of interest and background. Simple methods like thresholding do not work because they are not robust to noise and artifacts of images as well as images with overlapping cells [16]. Methods such as region growing, watershed, clustering and active contours have been attempted on cellular images [17-19]. However, these methods fail on images composed of overlapping or clustered cells. Cell segmentation is crucial to this work since tracking and subsequent analyses depend on the segmentation results. In our analyses, active contour without edges was used since it is not dependent on initialization, noise and boundary leakage by using intensity gradients [20,21]. The energy functional for regularization term is controlled by the length terms only and it was set according to the resolution of fluorescence intensity. The two phase level-set method is able to identify cells with maximum shape information since it handles sharp corners and cusps of the objects well. Thus, the original shapes of cells are retained yielding accurate features. Since dynamics of cell is dependent on geometric centroid, cell shape has to be accurately segmented. We subjectively evaluated segmentation results from different methods and confirmed that slight changes in the methods could dislodge the cell boundary by several pixels but did not affect the global boundary movement. Since we used run length of the boundary, minor boundary displacement did not affect the overall downstream analysis.

The spatiotemporal tracking method does not assume overlapping of cell boundaries between adjacent frames. It is able to handle dividing cells by using a set of heuristics. Four different scenarios are encountered during matching: (i) a cell in the current frame could match a cell in the proceeding frame (a successful match), (ii) no matching for cells in both frames (cells moving out of focus), (iii) one cell in current frame matches with more than one cell in the proceeding frame (possible cell division), and finally (iv) more than one cell in the current frame matches with only one cell in the proceeding frame (over segmentation). For differentiating case (iii) and case (iv), matching between second and third frame are checked to see whether a cell in multiple matches has its own unique characteristics. If a cell matches its counterparts in second and third frame, then we conclude that this cell has divided in the middle frame. If it has only one match in the third frame, then we conclude that this cell might have been over-segmented in the second frame. We used the same settings for weights as suggested by authors [22].

### Classification of cell features

**Figure 1:**
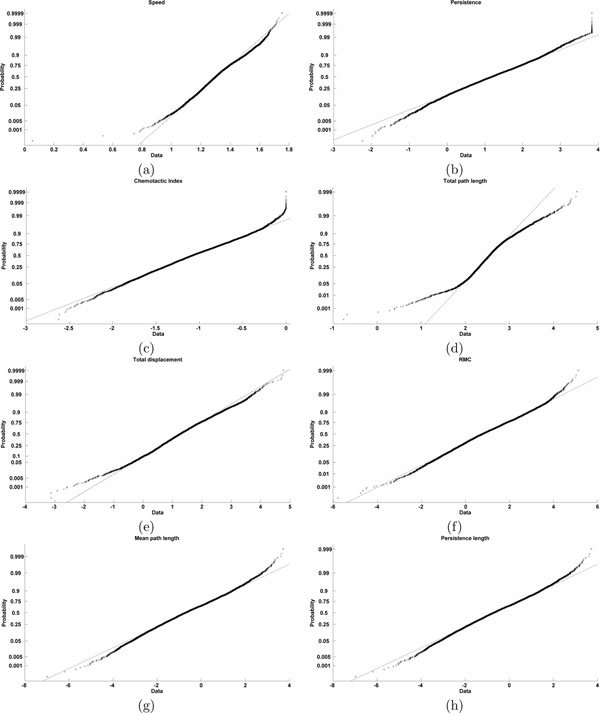
Tests of normality of features: every cross in the plot corresponds to midpoint in the jump of empirical cumulative distribution function on Y axis to sorted data in X axis (number of cells=5415).

The classification of cells is done in two steps. First, the numbers of clusters are found by modeling features by using a Gaussian mixture model; second, unsupervised K-means clustering was used with the number of clusters, obtained with GMM model. Since underlying structure of distribution of cell features are unknown, unsupervised approaches are more suitable. However, K-means clustering requires to know the number of clusters a priori. In order to implement Gaussian mixture models, cell features have to be normally distributed. We used probability plots and chi-square goodness-of-fit to test for Gaussianity of features. Probability plot is a graphical method for determining whether sample data conforms to a hypothesized distribution decided upon visual examination. The data is sorted and plotted against the midpoint in the jump of the empirical cumulative distribution function (CDF) on Y-axis. The CDF *F*(*z*), describes the probability that a random variable *z* with a given probability distribution takes on a value less than or equal to a specific value. The midpoint is given by (*j* – 0.5)/*N* for *j*th sorted value from a sample size of *N*. This plot also includes a reference line joining the first and third quartile and extrapolated out to the ends, which is useful for judging whether data follows a normal distribution. A departure from normality is indicated by presence of points away from the reference line (Fig. 1). All features except one, the total path length conformed to normality test. Since the cluster membership did not change significantly by removing total path length, this feature was included to derive the cell classes. A chi-square goodness-of-fit test also showed that Gaussian mixture modeling is appropriate to represent heterogeneous cell populations (Table 1).

**Table 1:** Chi-square goodness-of-fit for dynamic features

Feature	χ^2^	p value
Speed	2.62	< 0.001
Persistence	2.19	< 0.001
Chemotactic Index	9.92	< 0.001
Total path length	43.10	0.78
Total displacement	7.53	< 0.001
Random motility coefficient	7.07	< 0.001
Mean path length	4.40	< 0.001
Persistence length	4.40	< 0.001

The GMM model was computed for every possible number of subpopulations in the dataset (*K* = 2, 3, … 100). To eliminate the influence of convergence failures, each run was attempted up to 5 times with new initial conditions until convergence was reached. MDL criteria were used since it can lead to a consistent estimator even for large values of observations. For each value of *K*, MDL was calculated after convergence in the EM step. The optimal value of K corresponds to minimum MDL. In our dataset, this method identified four distinct subclasses from dynamic features. These classes were termed as (i) Class 1, (ii) Class 2, (iii) Class 3, and (iv) Class 4. The features for individual classes are tabulated in Table 2.

**Table 2:** Feature values of individual clusters

Feature	Class 1	Class 2	Class 3	Class 4
Speed (*µm*^2^*/h*)	9.50	9.53	11.95	12.32
Persistence (h)	1.46	8.93	10.57	0.82
Chemotactic Index	0.10	0.41	0.34	0.34
Total path length (*µm*)	15.07	14.46	16.89	17.27
Total displacement (*µm*)	1.85	6.10	6.55	6.04
Random motility coefficient	2.32	8.30	56.76	21.96
Mean path length (*µm*)	0.30	1.50	3.41	1.98
Persistence length (*µm*)	13.87	85.1	126.31	10.10

### Classification of edge features

**Figure 2:**
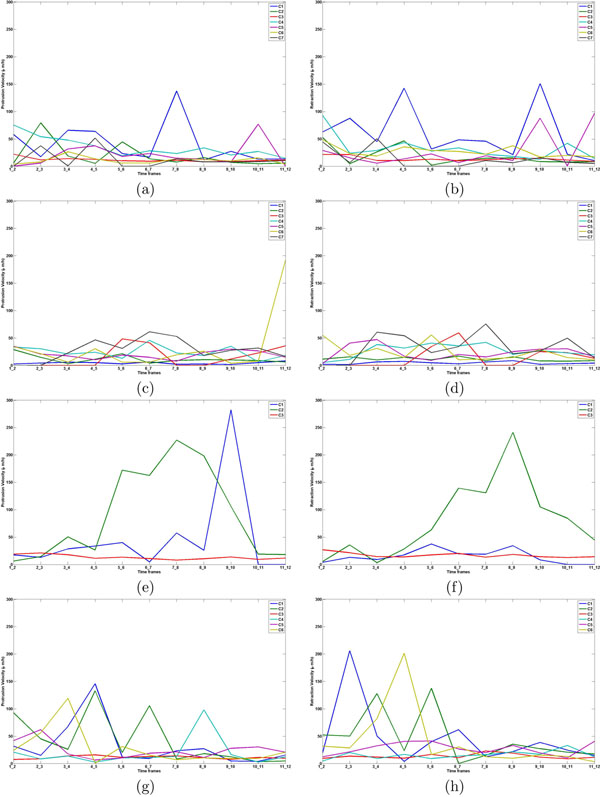
Edge prints for reference population from edge classes: left panel shows protrusion activity and right panel shows retraction activity for: class 1 (a and b); class 2 (c and d); class 3 (e and f); class 4 (g and h). The lines in each subplot represent edge prints of reference cells of each edge class.

The cell images sampled at 12 different time points provided a vector of values of protraction and retraction velocities respectively. This vector constitutes to an edge print of a cell, characterizing the membrane movement of the cell over time. For dataset with 12 time points, the features are computed using the adjacent frames. Finally we get a feature set of 11 protrusion features and 11 retraction features and thus 22 features in total. This set of measurements provides novel dynamic features to capture individual cell movements and membrane (edge) dynamics. This measurement does not necessarily inform about cell migration, since membrane retraction and protrusion without translocation can lead to high values. Reference sets for each cell class were estimated by K-means clustering. The initial centroids for K-means were obtained by performing the clustering phase on a random 10% sample of the data. Since the choice of initial cluster centroids is important, only 10% of randomly sampled data was used for K-means clustering. The centroids obtained from the subsamples (first phase) was used as seeds in the clusters for the second phase which used all the data. This procedure overcomes the problem of initialization in K-means clustering. About 1000 iterations were used each time to get the cluster centroids and members. K-means identified different number of sub-clusters in each of the cell classes (Fig. 2a - 2h).

### Correlation of cell and edge features

**Figure 3:**
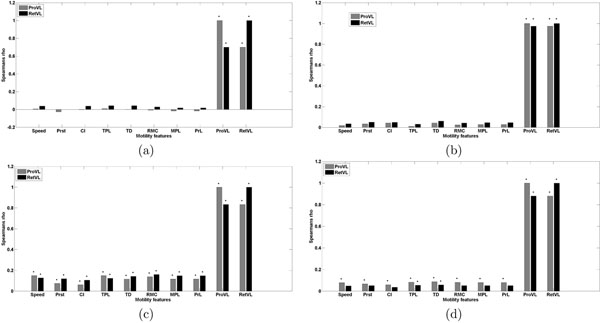
Correlation analysis of dynamic and edge features: Spearman rank correlation coefficient (*ρ*) demonstrates various levels of correlation among the features in different dynamic classes. The subplots (a), (b), (c), and (d) depicts distribution of features in four dynamic classes obtained from GMM clustering (class 1, class 2, class 3, and class 4). A black bullet on top of the bar represents significant correlation at (*p* < 0.05). CI: chemotactic index, TPL: total path length, TD: total displacement, RMC: random motility coefficient, MPL: mean path length, PrL: persistence length, ProVL: protrusion velocity, RetVL: retraction velocity.

To evaluate correlation between cell and edge features, Spearman’s rank correlation (*ρ*) and multiple correlation analysis (*R*^2^) were used on averaged dynamic and edge features over time. The Spearman rank correlation is a non-parametric measure of statistical dependence between two features using the ranks of features and is less sensitive to outliers. For this analysis, MATLAB function ‘corrcoef’ with type ‘Spearman’ was used. Correlation coefficient was computed for every pair of motility and edge features and the results were reported for statistically significant correlations at *p* < 0.05. The p-values were computed by transforming the correlation to create a t-statistic (; where *ρ* = correlation coefficient, *N* = number of samples) having *N* – 2 degrees of freedom and under the assumption that features are normally distributed. Rank correlations indicated that both motility and edge features varied in the degree of their correlation among clusters of cell dynamics (Fig. 3). The correlation plots in Figs. 3a - 3d show that the level of correlation varies among different classes. Multiple correlation measures the goodness-of-fit in linear regression; the ‘speed’ was the dependent variable and all other features (motility and edge features) were the predictors in regression analysis. This analysis showed strong positive correlation for all the features (*R^2^* = 0.97). In order to account for bias due to outliers in the regression analysis, we also performed jackknife cross-validation (results are given in Table 3). This qualitatively prove the existence of correlation of edge patterns with whole cell motility in individual classes.

**Table 3:** Leave-one-out cross-validation of correlation (mean ± std.dev) ×10^–4^

	Feature	Protrusion	Retraction
	Speed	55.78 ± 2.93	380.18 ± 3.20
	Persistence	–263.96 ± 0.40	–5.33 ± 0.45
	CI	–31.24 ± 1.98	377.82 ± 3.08
Class 1	TPL	95.82 ± 4.64	418.90 ± 3.66
	TD	15.80 ± 2.52	426.21 ± 2.11
	RMC	–88.32 ± 2.22	285.68 ± 1.60
	MPL	–168.86 ± 1.33	169.92 ± 3.51
	PrL	–169.26 ± 1.33	169.84 ± 3.51

	Speed	182.02 ± 1.15	346.26 ± 0.80
	Persistence	329.58 ± 0.65	498.53 ± 0.82
	CI	411.92 ± 3.50	489.53 ± 1.28
Class 2	TPL	84.41 ± 0.02	299.57 ± 0.48
	TD	427.14 ± 1.57	602.57 ± 0.80
	RMC	242.12 ± 2.11	413.72 ± 2.78
	MPL	280.53 ± 1.41	452.87 ± 1.82
	PrL	280.41 ± 1.41	452.79 ± 1.82

	Speed	149.57 ± 2.49	127.09 ± 1.82
	Persistence	736.33 ± 0.53	119.63 ± 0.96
	CI	599.60 ± 0.12	105.90 ± 0.28
Class 3	TPL	148.64 ± 1.62	123.50 ± 1.37
	TD	114.32 ± 0.63	142.40 ± 1.23
	RMC	137.66 ± 1.47	159.82 ± 1.26
	MPL	116.66 ± 3.48	148.01 ± 2.52
	PrL	116.66 ± 3.48	148.01 ± 2.52

	Speed	776.22 ± 0.01	481.55 ± 0.36
	Persistence	655.13 ± 0.61	504.45 ± 0.44
	CI	595.36 ± 0.40	360.12 ± 0.55
Class 4	TPL	828.03 ± 1.53	539.72 ± 0.20
	TD	872.38 ± 0.40	562.92 ± 0.40
	RMC	808.41 ± 0.43	506.85 ± 0.31
	MPL	796.51 ± 0.19	503.66 ± 0.23
	PrL	776.57 ± 0.19	503.71 ± 0.23

• Class 1: This class consists of cells with low speed and persistence. The pattern shows that active membrane ruffling may not translate into active cell movement. It might have even restricted the cells overall movement which is evident from the low total displacement feature. For example, NRK49F cells with defect in *rho* or adducin have been shown to have active lamellopodial ruffling, while being unable to migrate [23] (Fig. 2a and 2b).

• Class 2: Cells with medium speed and persistence showing positive correlation for protrusion and retraction. Similar protrusion and high retraction activity may be the reason for multiple peaks of edge features over the length of time (Fig. 2c and 2d).

• Class 3: This class is represented by fast moving cells displaying high speed and persistence and is positively correlated with edge movement features. These cells also had the highest edge activity which may help in moving the cell over long distances with high persistence. When the static features of these cells were analyzed they had typical fan shaped morphology (Fig. 2e and 2f).

• Class 4: These cells frequently change directionality as indicated by low persistence. Edge features are also positively correlated to dynamic features and this suggests that the frequent change in direction may be accompanied by a respective change in edge movements. Although the cells change direction, they travel within a limited radius more like in spiral motion. This can be seen from the low total displacement and mean path length compared to class 3. Even though, the cell speed is greater than Class 3, the cells do not travel in a constant direction (as indicated by low persistence) and tend to display a spiral or circular concentric motion (Fig. 2g and 2h).

In order to determine which features contributed to the diversity of correlation patterns, or rather influenced the type of motility pattern adapted by any cell, factor analysis was performed on all four sub-clusters. This method has been proven efficient in describing cell shape dynamics in cancer cells [24]. This method postulates the existence of a small number of latent factors which explains the systematic contribution of the original features. The number of factors that should be retained is suggested by the Kaiser criterion (factors with Eigenvalues more than or equal to one should be retained) [25]. For class 1 and class 2, six factors were retained which accounted for 91.6% and 90.1% of the variance respectively. For class 3 and class 4, seven factors were retained and they accounted for 88.2% and 89.0% of the variance respectively (Table 4). Factor 1 indicated the presence of high number of edge features. In particular, protrusion and retraction features extracted from initial six time points (Table 5 ). Factor 2 had predominantly cell dynamics features. The remaining factors contained edge features sampled from middle to end time points. These findings conclude that the motility patterns are decided largely by cell membrane features observed in the initial time points.

**Table 4:** Factor analysis on cell and edge features

	Class 1		Class 2		Class 3		Class 4	
	
Factor name (number)	Var	Cum. Var	Var	Cum.Var	Var	Cum.Var	Var	Cum.Var
Initial edge features (1)	35.69	35.69	35.08	35.08	38.64	38.64	24.92	24.92
Motility features (2)	24.39	60.06	22.02	57.28	17.79	56.44	20.58	45.50
Intermediate/late edge features (3)	10.19	70.28	13.80	71.09	11.12	67.56	11.90	57.4
Late retractions (4)	9.44	79.73	7.83	78.93	7.13	74.70	10.62	68.03
Intermediate retractions (5)	6.32	86.05	7.76	86.70	6.08	80.79	10.45	78.48
Intermediate retractions (6)	5.59	91.65	3.47	90.17	3.99	84.79	5.87	84.36
Late protrusions (7)	-	-	-	-	3.42	88.21	4.68	89.04

**Table 5:** Factor loading matrix computed from covariance matrix for all classes

Feature	Factor 1	Factor 2	Factor 3	Factor 4	Factor 5	Factor 6	Factor 7
Speed		-0.79					
Persistence		0.82					
CI		0.56					
TPL		-0.91					
TD		-0.73					
RMC		0.87					
MPL		0.96					
PrL		0.96					
*p*_1,2_	0.82						
*p*_2,3_	0.56						
*p*_3,4_	-0.80						
*p*_4,5_	0.68						
*p*_5,6_	-0.96						
*p*_6,7_	0.88						
*p*_7,8_			-0.93				
*p*_8,9_			-0.93				
*p*_9,10_							-0.45
*p*_10,11_			-0.85				
*p*_11,12_							0.83
*r*_1,2_	0.40						
*r*_2,3_	0.84						
*r*_3,4_	0.80						
*r*_4,5_	-0.91						
*r*_5,6_	0.75						
*r*_6,7_			-0.91				
*r*_7,8_						-0.91	
*r*_8,9_					-0.96		
*r*_9,10_			-0.69				
*r*_10,11_			-0.87				
*r*_11,12_				-0.79			

## Conclusion

Non-genetic heterogeneity in cell populations arises from a combination of intrinsic and extrinsic factors. This heterogeneity has been measured for gene transcription, phosphorylation, cell morphology, drug perturbations, and used to explain various aspects of cellular physiology. Our understanding of individual players in cell migration process is increasing; but there remains a vital gap to be filled concerning how they are coordinated spatially and temporally. New techniques are needed which can quantify dynamic cell movements at the level of single cell resolution in an automated manner.

Here, we report multivariate analysis of different sets of motility features through a meaningful combination of both novel (edge) and existing (centroid based) dynamic features. The first set of measurements has been already proved to improve subpopulation analysis. The second set of features is a novel measurement of edge activity. These features capture pixel movement, either through protrusion or retraction frame by frame over the entire length of observation. Since these measurements are temporally sampled, it is suitable to study cell activity over time. These features are unique and not necessarily a measurement of cell migration, as membrane protrusion-retraction is possible without translocation. Our data indicate different levels of correlation between sets of features, depending on the dynamic classes they belong to. This type of relationship was expected for this cell line due to its highly motile nature. Our findings compare well with previous literature [23].

The introduction of edge features is the major contribution of this work since it captures edge activity of large number of cells from high throughput imaging platforms in a way that no other profiling methods we are aware of have previously demonstrated. Our profiling method was able to provide additional insights which might have been missed using population based cell migration techniques or classical motility assays. To conclude, we have identified heterogeneous edge patterns of related dynamic profiles and validated our correlation patterns by comparing with previous publications. The dynamic profiles were obtained from cell displacement data by GMM clustering. Edge prints from these subclasses were further used to characterize heterogeneity arising due to different edge movements. The patterns arising from statistical correlation analysis were validated by comparing with previous publications. We also provided statistical evidence that initial time point edge features influence the motility patterns that a cell adapts.

## Methods

### Segmentation and tracking of cells

Level-set was used to segment cells from images, independently at all the time frames [20]. The image gradient was used to stop the evolution of level-sets. Touching cells were further separated by a marker-controlled watershed that uses initially segmented cells as shape markers for marking function [26]. The segmented cells in adjacent frames were correspondingly matched by spatiotemporal matching scheme that uses features like size, intensity, and spatial coordinates for matching [22]. The tracks of cells were subsequently corrected for mismatches and only those cells moving for the entire period of observation were included for further analysis.

### Dynamic feature extraction

Dynamic features of cells are classified into two categories based on motility modes: features describing whole cell dynamics and features representing membrane (edge) dynamics. Two different methods were employed to extract the two sets of features.

#### Cell dynamics

**Figure 4:**
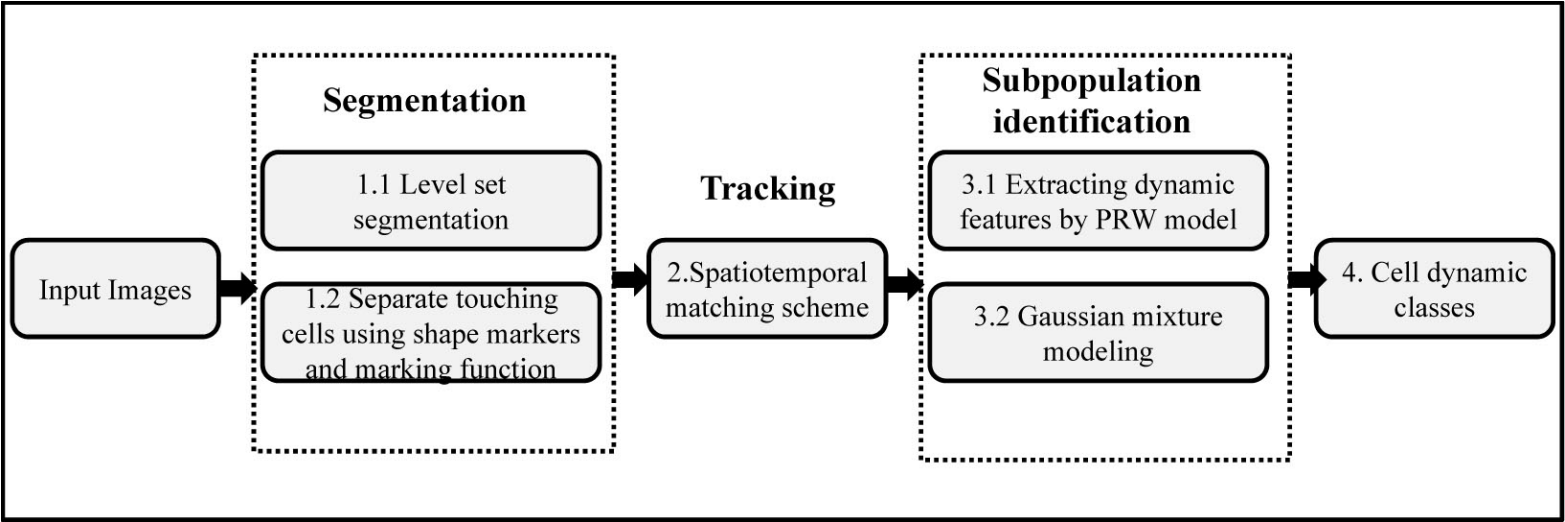
IIllustration of subpopulation identification using cell dynamics features: the time-lapse images are segmented by level-set framework followed by marker controlled watershed to separate touching cells; tracking by spatiotemporal scheme, clustering (GMM followed by K-means, and analysis of correlation among features).

A persistent random walk model was used to study directional migration of cells, in which the geometric centroid of a cell forms the basis for modeling cell motility [27]. A total of eight cell dynamics features were extracted: speed, persistence, chemotactic index (CI), total path length (TPL), total displacement (TD), random motility coefficient (RMC), mean path length (MPL) and persistence length (PrL) [3]. The set of subpopulations obtained from these features represent cell classes. The overview of the analysis is illustrated in the flowchart of Fig. 4 and Algorithm 1 summarises the different steps in the analysis.

A Gaussian mixture model (GMM) is used to represent feature distribution of the cell classes. The initial subpopulations were obtained by Gaussian mixture modeling of the cell feature distribution where each cluster is represented by a parametric distribution. The weighted sum of *K* component Gaussian densities is given by:

where  is a set of *N* samples and *x_i_* is the *i*th sample comprising of *n* features,  are the mixture weights, and  are component Gaussian densities. Each class density is a *n*-variate Gaussian function. The mixture weights satisfy the constraints that . The complete Gaussian mixture model is parameterized by the mean vector, covariance matrices and mixture weights from all component densities. These parameters are collectively represented as  where (*µ_k_*, Σ*_k_*) denotes the mean and covariance of the *k*th component.

Given training vectors and a GMM configuration, the parameters of GMM are given by maximum likelihood (ML) estimates .

ML estimates of parameters are obtained by using Expectation Maximization (EM) algorithm. In order to find the optimal number of classes, a minimum description length (MDL) estimator was employed [28]. MDL is an information theoretic model selection principle presumed as the most compact representation of data in the probabilistic network. MDL estimator finds the model order  by the following criteria:

Where . The penalty term in MDL includes the total number of features to avoid over-fitting of the model.

#### Edge dynamics

**Figure 5:**
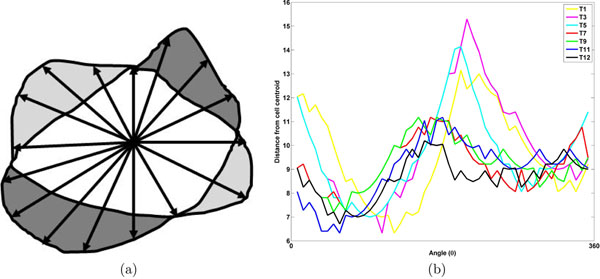
Illustration of edge feature extraction: (a) measuring protrusion or retraction displacements: heavy gray represents the protruded region while the light gray region represents retracted region, and (b) edge activity along the whole periphery, difference in radial length represented in Y axis and angle *θ* is represented in X axis.

Cell membrane features are defined as features characterizing movements of cell protrusions and retractions. Given a sequence of cell boundaries at the image frames, cells are aligned using their centroids. Edge pixels are then transformed to polar coordinates from Cartesian coordinates and a set of *M* markers  are placed on the segmented boundary *ϕ* of the cell marked by the radial coordinate. The movement of cell boundary *ϕ_t_* at time *t* to *ϕ_t_*_+_*_τ_* at time *t* + τ is calculated by measuring the displacements of individual markers within an interval τ. Protrusion and retraction features  of a cell are computed as a function of marker displacements over sampling intervals τ. A positive displacement is considered as a protrusion and negative displacement a retraction. The protrusion and retraction features are computed from total boundary displacement *ν*(*t* : *τ*) of the cell at time *t*:

where *ϕ*_*m*,*t*_ denotes the location of the *m*th marker of the boundary *ϕ_t_* at time *t*. The protrusion *p_t_* and retraction *r_t_* features at each time point *t* are then computed and features are extracted thereof. Fig. 5 illustrates the steps involved in evaluating edge features.

Cells are classified by a set of protrusion and retraction features measured over all the time points. These features provide an idea about the activity level of a cell at respective time instances and are used to cluster the cells. Clustering was performed using K-means algorithm.

## Competing interests

The authors declare that they have no competing interests.

## Authors contributions

MV conceived, designed and wrote the paper. RW participated in the design of study and helped draft the manuscript. AN performed the wet-lab experiments and collected the data, PM designed the wet-lab experiments, JCR conceived, designed and helped write paper. All authors read and approved the final manuscript.
